# Designs of studies published in two Brazilian journals of orthopedics and sports medicine, recently indexed in the ISI Web of Science

**DOI:** 10.1590/S1516-31802009000600007

**Published:** 2010-05-21

**Authors:** Rachel Riera

**Affiliations:** I MD, MSc. Research assistant in the Brazilian Cochrane Center and Attending Physician in the Discipline of Emergency and Evidence-Based Medicine, Universidade Federal de São Paulo - Escola Paulista de Medicina (Unifesp-EPM), São Paulo, Brazil.

**Keywords:** Journal impact factor, Methods, Periodicals as topic, Research design, Publications, Fator de impacto de revistas, Métodos, Publicações periódicas como assunto, Projetos de pesquisa, Publicações

## Abstract

**CONTEXT AND OBJECTIVE:**

The methodology and relevance of articles are among the keystones for promoting their citation and increasing journals’ impact factors. Study designs appropriate for answering the questions and adequate sample sizes have the aim of reducing the risk of bias. This study evaluated the articles published in two Brazilian journals of orthopedics and sports medicine that were recently indexed in the ISI Web of Science, regarding study design, sample size calculation, randomization and blinding.

**DESIGN AND SETTING:**

Descriptive study at Brazilian Cochrane Center.

**METHODS:**

Through a manual search, all original manuscripts published in 2007 in Acta Ortopédica Brasileira and Revista Brasileira de Medicina do Esporte were selected and evaluated.

**RESULTS:**

All the 60 articles published in Acta Ortopédica Brasileira and the 87 articles in Revista Brasileira de Medicina do Esporte were included and evaluated. The commonest design in Acta Ortopédica Brasileira was experimental studies (n = 19) and in Revista Brasileira de Medicina do Esporte, update or review articles (n =14). Sample calculations were seen in a minority of the articles. None of the eight clinical trials published presented sample calculations or adequate randomization processes. Three were described as blinded, but none described the measures taken to prevent disclosure of the allocation concealment.

**CONCLUSIONS:**

Publication of studies of good methodological quality other than review and experimental studies should be strongly encouraged among Brazilian journals, with the aim of increasing their citation and therefore their impact factor.

## RESUMO

**CONTEXTO E OBJETIVO:**

A qualidade metodológica e a pertinência dos artigos estão entre algumas das peças fundamentais para promover sua citação e aumentar o fator de impacto da revista. Um desenho de estudo apropriado para responder às perguntas e uma amostra de tamanho adequado visam reduzir o risco de viés. Este estudo avaliou os artigos publicados em duas revistas brasileiras das áreas de ortopedia e medicina esportiva recentemente indexadas no ISI Web of Science, com relação a desenho do estudo, cálculo do tamanho amostral, aleatorização e mascaramento.

**TIPO DE ESTUDO E LOCAL:**

Estudo descritivo no Centro Cochrane do Brasil.

**MÉTODOS:**

Por meio de pesquisa manual, foram selecionados e avaliados todos os manuscritos originais publicados em 2007 na Acta Ortopédica Brasileira e Revista Brasileira de Medicina do Esporte.

**RESULTADOS:**

Todos os 60 artigos publicados na Acta Ortopédica Brasileira e os 87 artigos da Revista Brasileira de Medicina do Esporte foram incluídos e avaliados. O desenho mais comum na Acta Ortopédica Brasileira foi de estudo experimental (n = 19) e na Revista Brasileira de Medicina do Esporte, de atualização ou revisão (n = 14). O cálculo da amostra foi observado em uma minoria dos artigos. Nenhum dos oito ensaios clínicos publicados apresentou o cálculo do tamanho da amostra, nem o processo de aleatorização adequado. Três foram descritos como cegos, no entanto, nenhum descreveu as medidas tomadas para impedir a quebra do sigilo da alocação.

**CONCLUSÕES:**

A publicação de estudos de boa qualidade metodológica, que não estudos experimentais e de revisão, deve ser fortemente incentivada entre os periódicos brasileiros com o objetivo de aumentar a sua citação e, portanto, seu fator de impacto.

## INTRODUCTION

The impact factor is a tool for ranking, assessing and comparing scientific journals. It is a measure of the frequency with which the “average article” in a journal has been cited in a particular year or period. It is generated from the citation reports in the Science Citation Index (SCI) and it is calculated by dividing the number of current-year citations of the source items published in that journal by the number of articles published during the previous two years. The Institute for Scientific Information (ISI), now called Thomson Scientific, is the abstracting and indexing company that publishes the Science Citation Index (SCI), Journal Citation Reports (JCR) and Social Sciences Citation Index (SSCI).^1^

Recently, eight Brazilian medical journals were included in the Journal Citation Reports database and will have their first impact factor available from 2010 onwards: Acta Ortopédica Brasileira, Arquivos Brasileiros de Cardiologia, Clinics, Journal of Pediatry, Jornal de Pediatria, Revista Brasileira de Cirurgia Cardiovascular, Revista Brasileira de Medicina do Esporte, Revista da Associação Médica Brasileira and São Paulo Medical Journal.

The methodological quality and relevance of articles are among the keystones for promoting their citation and increasing journals’ impact factors. Choosing a study design appropriate for answering each question type has the aim of reducing the risk of bias. Similarly, the sample calculation and the description of how it was done present readers with information on the risks of type 1 (alpha) and type 2 (beta) errors. Thus, in the present study, we evaluated the study design, presence of the sample size calculation and the frequency of the use of randomization and blinding among the clinical trials published in two Brazilian medical journals.

## OBJECTIVES

To evaluate the articles in two Brazilian journals within the fields of orthopedics and sports medicine that were recently indexed in the ISI Web of Science, for the following outcomes: a) study design, b) frequency of descriptions of the sample size calculation and c) frequency of the use of randomization and blinding, among the clinical trials.

## METHODS

Through a manual search, all manuscripts classified as original that were published in Acta Ortopédica Brasileira (AOB) and Revista Brasileira de Medicina do Esporte (RBME) in 2007 were selected and evaluated. To evaluate the sample size calculation, experimental studies on animals or corpses, validation studies on questionnaires, qualitative studies, editorials, narrative reviews or updating articles, letters to the editor and articles included in sections called Case Reports or Case Series were excluded. To estimate the frequency of the study design, these exclusion criteria were not applied and all articles were considered.

The articles were classified according to the purpose of the study (treatment, prevention, diagnosis, prevalence, incidence, prognosis etc) and the study design (systematic review, randomized clinical trial, cohort study, prospective cohort, case-control, cross-sectional prevalence or accuracy).^2^

The frequency of the presence of sample size calculations and the frequency of the use of randomization and blinding were also evaluated as outcomes.

## RESULTS

We evaluated all articles published in the two journals in 2007 that met the inclusion criteria. Acta Ortopédica Brasileira released five issues containing 60 articles, but to estimate the frequency of sample size calculations, the exclusion criteria were applied and only 30 articles were assessed. Revista Brasileira de Medicina do Esporte published 87 articles in six issues in 2007 and after applying the exclusion criteria for estimating the frequency of sample size calculations, only 52 articles were considered (Table 1).

The distribution of the selected articles according to the clinical question evaluated is presented in Table 2. The distribution of the selected articles in relation to the study design and the frequency of sample size calculations among the articles are presented in Table 3 and Table 4, respectively. Table 5 ^3^^,^^4^^,^^5^^,^^6^^,^^7^^,^^8^^,^^9^^,^^10^ presents the characteristics of the eight clinical trials published in these journals.

**Table 1.** Distribution of all published articles

Published articles	*Acta Ortop Bras*	Revista Bras Med Esporte
	n (%)	n (%)
Articles that were assessed for the outcome “study design” (before applying exclusion criteria)	60 (100)	87 (100)
Articles excluded from the assessment of the outcome “frequency of sample size calculation”	30 (50)	35 (40.2)
In Review or Updating section	4 (6.6)	14 (16)
In Case Series or Case Report section	4 (6.6)	1 (1.1)
Validation Study	3 (5)	9 (10.3)
Experimental Study on animals or corpses	19 (31.6)	7 (8)
Qualitative Study	0	1 (1.1)
Editorial	0	3 (3.4)
Remaining articles, which were assessed for the outcome “frequency of sample size calculation”	30 (50)	52 (59.8)

**Table 2.** Distribution of the selected articles regarding the clinical

Clinical question	***Acta Ortop Bras*, n (%)**	Revista Bras Med Esporte, n (%)
Incidence	2	0
Diagnosis	2	3
Prevalence/frequency	13	32
Prevention	0	0
Prognosis/risk factor	4	0
Treatment/effect	9	17
Total	30 (100%)	52 (100%)

**Table 3.** Distribution of the selected articles regarding the study

Design	Acta Ortop Bras, n (%)	Revista Bras Med Esporte, n (%)
Accuracy	0	0
Case-control	0	0
Prospective cohort	2 (6.6)	11 (21.1)
Retrospective cohort or case series	8 (26.6)	0
Clinical trial	3 (10)	5 (9.6)
Systematic review	0	0
Cross-sectional prevalence	15 (50)	32 (61.5)
Analytical cross-sectional	2 (6.6)	4 (7.7)
Total	30 (100)	52 (100)

**Table 4.** Distribution of the articles in accordance with the frequency of sample size calculation

	Acta Ortop Bras n (%)	Revista Bras Med Esporte n (%)
Described the sample size calculation	2 (6.6)^3^^,^^4^	2 (3.8)^5^^,^^6^

**Table 5.** Characteristics of the clinical trials published

Study	Sample size calculation	Described as randomized	Randomization clarified	Described as double-blinded	Blinding clarified
Souza Júnior et al.^3^	No	Yes	No	Yes	No
Bertolla et al.^4^	No	Yes	No	Yes	No
Angeli et al.^5^	No	Yes	No	Yes	No
Trevisan and Burini^6^	No	No	-	No	-
Gama et al.^7^	No	Yes	No	No	-
Silva et al.^8^	No	No	-	No	-
Nascimento et al.^9^	No	Yes	Yes, but was inadequate	No	-
Benegas et al.^10^	No	Yes	No	No	-

## DISCUSSION

In addition to methodological quality, the relevance of studies for clinical practice and for future research is a key factor. Studies may be responsible for changing actions, influencing healthcare decision-makers and allocating research resources.

With rare exceptions, articles reporting experimental studies or narrative reviews are insufficient to justify or support healthcare decision-making, regarding questions about treatment, prevention or prevalence. However, we found a relatively large number of experimental studies on animals or corpses in Acta Ortopédica Brasileira (n = 19; 31.6%) and perhaps this finding can be explained by the fact that Orthopedics is a surgical specialty in which experimental studies are extremely necessary for the process of developing new interventions. On the other hand, there was also a significant number of reviews or updating articles in Revista Brasileira de Medicina do Esporte (n = 14; 16%).

For both journals, the most common clinical question addressed in the articles related to prevalence or frequency, probably because of the lower degree of difficulty and shorter time usually required to carry out cross-sectional studies that can answer these questions.

Furthermore, as expected and in accordance with the above, the study design most frequently found among the articles was the cross-sectional prevalence type. Approximately 10% of the original articles published in both journals were clinical trials, whereas no systematic reviews were published.^3^^,^^4^^,^^5^^,^^6^^,^^7^^,^^8^^,^^9^^,^^10^

It should be noted that only two articles in each journal presented sample calculations. In other words, among the studies published in 2007 (and after application of the exclusion criteria), sample size calculations were only found in 6.6% of the articles in AOB and 3.8% of the articles in RBME.^11^^,^^12^^,^^13^^,^^14^

One further important observation was that among the eight clinical trials published in the two journals, none of them presented data on the calculation of the sample size. This type of methodological weakness can compromise the reliability of the results and hence the applicability of the study in clinical practice.

With regard to randomization, among the six studies described as randomized, there was no explanation of the randomization process in five of them.^3^^,^^4^^,^^5^^,^^6^^,^^7^^,^^8^^,^^10^ In one of them, the randomization process was performed by taking the last general hospital registration number on each patient’s medical files: those for whom the last digit was an even number were included in group A and those with an odd number were put in group B (quasi-randomization).^9^ According to Jadad, the presence of adequate randomization is among the main requirements for a clinical trial of good methodological quality.^15^

With regard to blinding, among the eight trials, three were described as blinded. However, in all of them, there was no description of the measures taken to prevent disclosure of the allocation concealment.

Among the 30 original articles published in AOB (after applying the exclusion criteria), ten related to surgical interventions.^9^^,^^10^^,^^11^^,^^16^^,^^17^^,^^18^^,^^19^^,^^20^^,^^21^^,^^22^ The difficulties in conducting trials on surgical interventions are well known and cannot be denied. However, once it is decided to conduct a randomized clinical trial, the sample size calculation, selection and description of the randomization and blinding of the researcher/evaluator and, whenever possible, of the patients, are required in order to provide evidence that can be safely incorporated into clinical practice.

In 2007, only one article reporting on qualitative research was published in these two Brazilian journals.^23^ The development and publication of articles other than review studies and experimental should be further encouraged among Brazilian journals, with the goal of providing high-level evidence that can be used to guide practical approaches, but also with the aim of increasing the methodological quality of the published articles, thereby increasing their citation and the journals’ impact factors.

## CONCLUSION

In 2007, the study design most commonly used in Acta Ortopédica Brasileira was experimental studies on animals or corpses, while in Revista Brasileira de Medicina do Esporte, it was updating or review articles. The presence of sample calculations was observed in a minority of the original articles in both journals. Among the eight clinical trials published, none presented this calculation and either there was no description of the randomization process or it was inadequate. In the three studies described as blinded clinical trials, there was no description of the measures taken to prevent disclosure of the allocation concealment. The publication of studies of good methodological quality other than reviews and experimental studies should be further encouraged among Brazilian journals, with the aim of increasing article citation and therefore the journals’ impact factors.
